# New Methods for Replacing Single Missing Teeth with Non-prep Bridges (NPBs) – A Case Series

**DOI:** 10.3290/j.jad.b4515555

**Published:** 2023-10-16

**Authors:** Hans Jörg Staehle

**Affiliations:** a Professor, Department of Conservative Dentistry, Clinic for Oral, Dental and Maxillofacial Diseases, University Hospital Heidelberg, Germany.

**Keywords:** all-composites, cantilever design, gap-closure in the posterior area, non-invasive restorative methods

## Abstract

**Purpose:**

Newly developed non-invasive methods for replace a missing tooth and closing single-tooth gaps in the posterior region using resin composite are presented.

**Materials and Methods:**

Four different non-invasive methods and the technical procedures, materials and instruments used are presented in a case series. These include the direct intraoral insertion of composite (with and without individual shaping aids) and indirect restorations, which are fabricated conventionally or digitally and bonded.

**Results:**

The case series showed that all four methods can be used to replace single missing teeth in the posterior region, meeting current clinical requirements. Particular attention was paid to the design of the pontics, the dimension of the connector area, firm proximal contacts to the adjacent teeth, hygiene, and appearance of the non-prep bridges (NPBs). The advantages and disadvantages for both the direct and indirect techniques illustrated in this case series were compared in detail.

**Conclusions:**

Several direct and indirect non-invasive methods for single-tooth replacement are available today. Although the evidence is still limited, there is a potential for frugal dental interventions with NPBs. Further experimental and clinical studies are necessary to demonstrate that they reliably meet quality requirements (including sufficient survival rates), satisfy the criteria of cost-effectiveness (compared to treatment alternatives) and that there is a demand from the population.

Today, implants and fixed partial dentures of varying invasiveness are used to close gaps in the posterior region. The current standard procedures in prosthetic dentistry require at least minimal preparation of the abutment teeth. Although implants can close single-tooth gaps without damaging the adjacent teeth, they are cost-intensive and require surgical measures.

To date, the only restorative procedures in the posterior region that have completely eliminated the need for preparation of abutment teeth are directly placed composite buildups and composite cantilever restorations.^9,10,12,15,19^ The techniques for the direct fabrication of the categories mentioned in Fig 1 have been presented in a number of case reports and a pilot study.^13,14,19^ It was shown that restorations in these categories are successful in principle, if special design requirements (e.g., selective oversizing of the palatal/lingual connector region to avoid fractures of the composite) are considered. However, the technical approach described so far is complex and therefore sometimes difficult to implement in daily practice. For this reason, modifications and further procedures have been developed, which are explained in more detail here.

**Fig 1:**
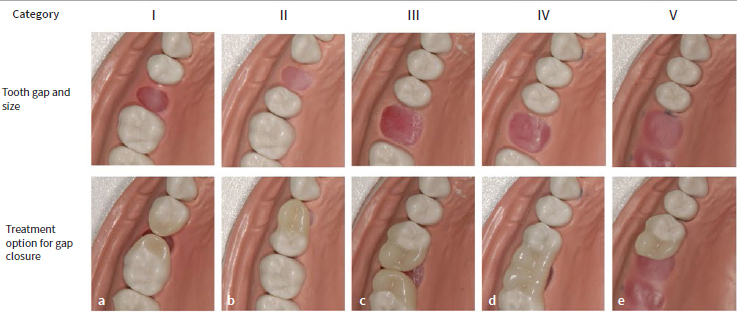
Range of options for non-invasive, metal- and fiberglass-free composite restorations (non-prep bridges) to close single tooth gaps in the posterior region. (a) Category I: two single-retainer composite buildups (interdental gap up to premolar width). (b) Category II: one single-retainer composite cantilever restoration (interdental gap up to premolar width). (c) Category III: two single-retainer composite cantilever restorations (interdental gap up to molar width). (d) Category IV: one double-retainer block connection (interdental gap up to molar width). (e) Category V: one single- retainer free-end composite cantilever restoration (for a free-end gap). Top row: initial situation in each case; bottom row: after restoration in each case.

## Materials and Methods

Among the non-invasive methods for replacing single missing teeth with composite materials, a main distinction can be made between a) direct intraoral insertion of composites and b) indirectly (extraorally) fabricated restorations:

- Direct intraoral insertion of composites has so far been performed in NPBs without individual shaping aids.^12^ A novel variant is the use of individual shaping aids (flexible splints). In this case, various requirements such as isolation of the working field, shaping of the restoration, and adhesive placement of the composite (including attachment to the abutment tooth) are fulfilled in a single working step. Regarding adhesive bonding, a direct and therefore “simple” interface (tooth surface/composite) is created.
- New types of indirectly fabricated restorations are either modeled directly on a plaster model outside of the oral cavity with composites or fabricated from blocks of resin composite or hybrid ceramic materials using CAD/CAM techniques. Special designs developed for these types of restorations are used. Adhesive bonding of indirect restorations creates a “double” interface (tooth surface/composite cement/restoration).

Common to all these procedures is that they feature the design of selective oversizing in the connector area (especially on the palatal or lingual side). This makes it possible to dispense with preparations of the abutment tooth altogether.^12^

An overview of the four methods can be found in Table 1. The materials used are listed in Table 2.

**Table 1:** Overview of procedures for non-prep bridges


**I. Direct procedures**
1. direct intraoral composite insertion without individual “shaping aids”
2. direct intraoral composite insertion with individual “shaping aids” (laboratory-made matrices or flexible silicone indices)
**II. Indirect procedures**
3. indirectly (extraorally) fabricated restorations (manual modeling)
4. indirectly (extraorally) fabricated restorations (digitally fabricated from composite or composite-ceramic combinations)

**Table 2:** List of the most important instruments and materials used

Material/Instrument	Manufacturer
Rubber-dam	Hygienic Dental Dam (heavy/medium), Coltene Whaledent; Altstätten, Switzerland
Rubber-dam ligation	Wedjet small (H06522), Coltene Whaledent
Adhesive	Methods 1 and 2: Optibond FL, Kerr; Orange, CA, USA Method 3 and 4: Panavia V5, Kuraray Noritake; Osaka, Japan
Flowable composite	Tetric Evo Flow; Ivoclar Vivadent; Schaan, Liechtenstein
Restorative resin composite	Tetric Prime; Ivoclar Vivadent
Modeling instrument	Optra Sculpt; Ivoclar Vivadent
Light curing	Units: Elipar S 10 (3M Oral Care; St Paul, MN, USA). Irradiance: 860 mW/cm^2^ Valo (Ultradent). Irradiance: 820 mW/cm^2^; irradiation duration – depending on layer thickness – at least 20 s up to 40 s
Scalpel blade	No. 12, No. 871B/12, Carl Martin; Solingen, Germany
Oscillating diamond instruments (excess removal)	Sonic Set shape SFD 2F, SFM 2F, SFD 4f, SFM, 4f; Endo-Sonic-Access SF 68, SF 69, Komet, Gebr. Brasseler; Lemgo, Germany
Interdental brushes	Curaden International, Kriens, Switzerland and Interprox plus, Dentaid, Cerdanyiola, Spain
Silicone splints	Fegura Sil Glass, Feguramed; Buchen, Germany
Digital workpiece production	Cerec, Dentsply Sirona; Bensheim, Germany
Milling blocks	Vita Enamic, Vita, Bad Säckingen, Germany*; Tetric CAD, Ivoclar Vivadent (used here)

*In the case of Vita Enamic, the ceramic should be etched with hydrofluoric acid in addition to the steps described above.

## Results

The technical procedures of the four methods mentioned, including the treatment results obtained, are presented below.

### Method 1: Direct Intraoral Composite Insertion without Individual Shaping Aids

**Fig 2:**
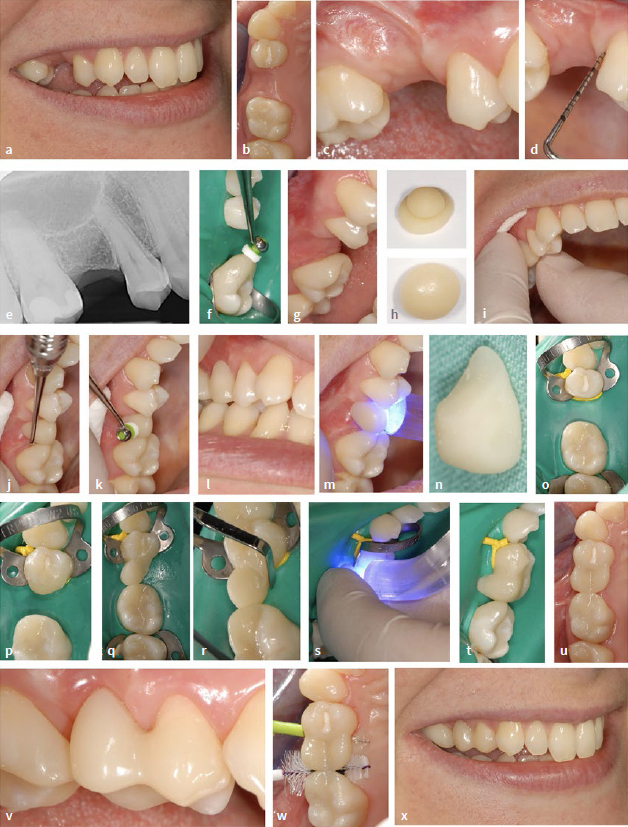
Direct intraoral composite insertion without individual shaping aids using the example of a category II NPB in a 31-year-old female patient. (a) to (c) Initial situation. (d) Periodontal probing (no increased probing depths, no bleeding). (e) Radiograph of the initial situation. (f) and (g) Mesial enlargment of the maxillary right first molar with directly applied composite. (h) Composite “ball” of dentin translucency on composite “plate” of enamel translucency (top) is formed into an ovoid shape with dentin composite in the core and enamel composite on the outside (bottom). (i) Careful insertion of the soft composite “egg”. (j) and (k) shaping of the composite. (l) in maximum intercuspidation. (m) Light curing. (n) Initial pontic after removal from the oral cavity. (o) Situation under rubber-dam. The abutment tooth is fitted with a rubber ligature which is pressed towards the gingiva with a rubber-dam clamp. Condition after etching with phosphoric acid, rinsing and drying. (p) After application of primer and adhesive, light curing and application of composite on the distal surface of the upper right first premolar (without light curing). (q) Insertion of the initial pontic. (r) Composite adaptation. (s) Light curing. (t) Application of further composite for final shaping. (u) and (v) after finishing and polishing. (w) Fitting of interdental brushes. (x) Treatment result.

The technical procedure for fabricating a composite cantilever restoration (category II, Fig 1) was described in detail in 2019.^12^ In the case described here, a composite buildup (Category I, Fig 1) was combined with a composite cantilever restoration (Category II, Fig 1). The method outlined in 2019 has been modified, specifically in the adjustment of the pontic’s design (see below for details). A 31-year-old female patient who had a persistent deciduous tooth extracted three years prior to initial presentation desired a non-invasive restoration to improve her appearance. The general medical history revealed a blood coagulation disorder (Willebrand-Jürgens type 1) and thyroid disease (Hashimoto’s disease). The clinical findings regarding dental hard tissues, existing restorations, endodontic, periodontal and functional parameters proved to be without pathology. Figures 2a to 2c show the initial situation with the gap in the region of the maxillary right second premolar. Periodontal probing did not reveal any increased probing depths or subsequent bleeding on the two abutment teeth (maxillary right first molar and first premolar) (Fig 2d). Figure 2e shows the initial radiographic situation.

#### Composite buildup of the maxillary right first molar

First, the maxillary right first molar was enlarged using directly placed resin composite according to the method described by Staehle^13^ to reduce the gap to premolar width (Figs 2f and 2g).

#### Composite cantilever restoration of the upper right first premolar

**Fig 3:**
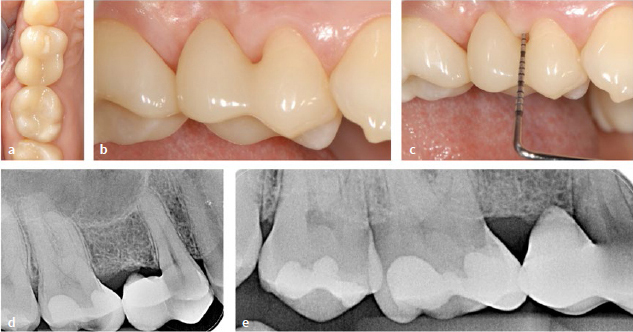
Same patient as in Fig 2, at the 1-year check-up. (a) to (c) inconspicuous clinical situation including periodontal conditions. (d) and (e) Radiographic situation.

Compared to the procedure described in 2019 for the fabrication of direct tooth attachments, there is now a modification in the fabrication of the pontic.^12^ The fabrication of the direct pontic was modified to the extent that rubber-dam application was initially omitted to match its basal design to the surface of the alveolar ridge. For this purpose, composite was applied directly to the alveolar ridge under relative isolation. To close the remaining gap completely, a small amount of resin composite (Tetric Prime, conventional packable, Ivoclar Vivadent; Schaan, Liechtenstein) with dentin translucency was placed on top of a larger, flattened portion of resin composite (also conventional packable) with enamel translucency (Fig 2h top), wrapped around it and shaped into an egg (Fig 2h bottom). If the proportions had been larger, the inner sphere could have been light cured extraorally in advance for sufficient polymerization. The “soft” (not yet light-cured) resin composite prepared in this way was carefully introduced into the gap and adapted with a Heidemann spatula and a modeling instrument (Optrasculpt, Ivoclar Vivadent), taking care, among other things, to ensure good shaping of the pontic base in contact to the alveolar ridge (Figs 2j and2 k). Oversizing in the connector area was initially avoided so that the initial pontic could be removed and inserted without difficulty. Occlusion was also checked (Fig 2l). Subsequently, light curing was performed (Elipar S10, 3M Oral Care; St Paul, MN, USA; irradiance 860 mW/cm^2^; irradiation time according to the layer thickness; ≥20 s up to 40 s in each of the occlusal, vestibular and palatal directions; see also Table 2) (Fig 2m). The pontic in its initial shape was removed (Fig 2n) and light cured again extraorally from all sides. Small irregularities were straightened and shaped with a scalpel. Subsequently, rubber-dam was applied, and a ligature was inserted on the abutment tooth, which was positioned gingivally with a rubber-dam clamp (Fig 2o). After etching with phosphoric acid, rinsing, drying, and primer and adhesive application (Optibond FL, Kerr; Orange, CA, USA) with subsequent light curing, some resin composite was applied to the distal surface of the maxillary right first premolar (Fig 2p). If other restorative surfaces need to be included due to preexisting restorations, additional working steps (e.g., air abrasion with aluminum oxide powder, material-specific bonding agents) might be necessary. However, this did not apply in the cases described (see below). The polymerized pontic in its initial form was pressed into the uncured composite, causing it to bulge (Fig 2q). Figure 2r shows the composite adaptation using a Heidemann spatula. This was followed by light curing (Fig 2s) and further application of composite (Fig 2 t). The composite was slightly oversized in the connector area buccally and greatly oversized palatally. Oscillating diamonds (Sonic Set shape and Endo-Sonic-Access, Komet Gebr. Brassler; Lemgo, Germany, see Table 2 for details) were required for finishing, especially at the transition to the abutment tooth. Figures 2u and 2v show the pontic after finishing and polishing. To ensure hygiene, suitable interdental brushes were selected which could be passed through the newly created interdental spaces with moderate insertion force (Fig 2w). Fig 2x shows the treatment result (compare with the initial situation in Fig 2a). The materials used can be found in the material list (Table 2). Figure 3 (same patient as in Fig 2) shows the condition at the one-year check-up. The findings (including periodontal parameters) are without pathology. The clinical situation is shown in Figs 3a to 3c, and the corresponding radiographs are shown in Figs 3d and 3e. The patient was satisfied with the result.

### Method 2: Direct Intraoral Composite Insertion with a Laboratory-made Individual Silicone Index

**Fig 4:**
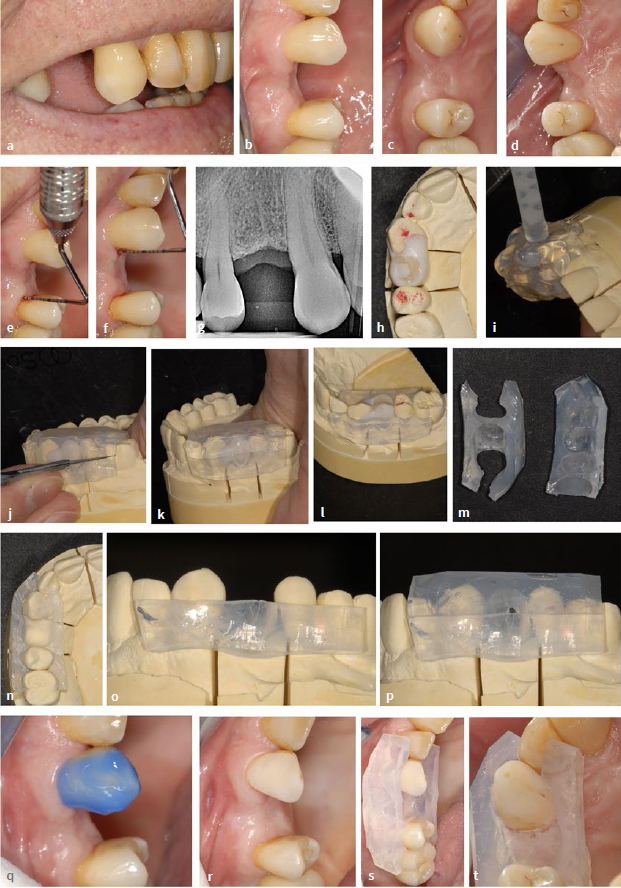
Direct intraoral composite insertion with horizontally split, individual shaping guides (splints, silicone indices) using the example of a category II NPB in an 81-year-old patient. (a) to (d) Initial clinical situation with gap in the region of the maxillary right first premolar. (e) and (f) Bleeding on probing. (g) Radiographic situation. (h) Model with wax-up. (i) to (m) Shaping, trimming and horizontal division of a flexible splint. (n) to (p) Check for accuracy of fit on the model. Note the drainage channel for composite (p). (q) and (r) Etching, rinsing and drying of the abutment tooth. (s) and (t) Inserting and testing the splint (silicone index) on the patient.

**Fig 5:**
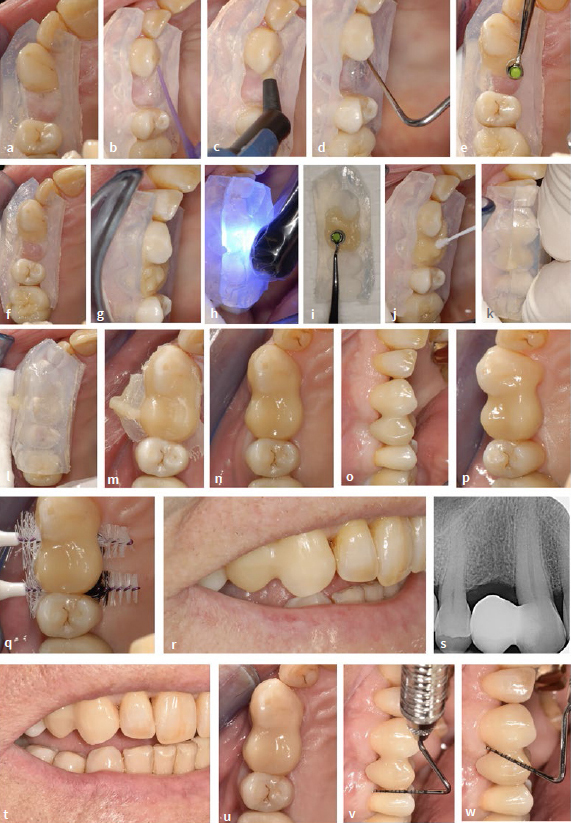
Same patient as in Fig 4. (a) After application of primer and adhesive according to manufacturer’s instructions on the abutment tooth. (b) Insertion of flow composite into the basal splint areas facing the abutment tooth; no initial light curing. (c) Insertion of restoration composite (dentin translucency). (d) Plugging of the restorative composite into the uncured flow composite (snowplow technique). (e) and (f) Adaptation of the restoration composite using a modeling instrument (Optrasculpt). (g) Successive composite build-up, buccal portion with enamel translucency, otherwise with dentin translucency. Mamellon-like shaping of the occlusal area. (h) Light-curing after insertion of the still empty coronal splint part. (i) Filling of the coronal splint part with restoration composite in enamel translucency. (j) Application of a small amount of flow composite. (k) and (l) Positioning and pressing down of the coronal splint part with subsequent light curing from all sides. (m) After removal of the splint. (n) to (p) After finishing and polishing. (q) Try-in of individually selected interdental brushes. (r) Extraoral image to evaluate the appearance. (s) Radiographic control. (t) to (w) at the ½ -year check-up. Inconspicuous clinical situation including periodontal conditions, disappearance of bleeding on probing.

In the case described here (Figs 4 and 5), a category II composite cantilever restoration (Fig 1) was fabricated using a flexible splint. The patient was 81 years old and wanted a gap closure due to the loss of the maxillary right first premolar. The patient was under bisphosphonate medication. The clinical situation is shown in Figs 4a to 4f. Figure 4g shows the situation in the dental radiograph. The clinical findings regarding dental hard tissues, existing restorations, endodontic, periodontal and functional parameters proved to be largely unremarkable. On probing, there was discrete bleeding on the teeth bordering the gap, but there were no increased probing depths (Fig 4e and 4f).

#### Fabrication of the silicone index

Plaster models of the maxilla and mandible were fabricated using alginate impressions. On the upper model, the alveolar ridge in the region of the maxillary right first premolar was reduced slightly in the area of the pontic. The shape of the pontic was designed with the aid of a prosthetic tooth fitted into the gap and adapted with modelling wax (Fig 4 g). The resulting situation was directly coated with vinyl polysiloxane material (Fig 4i), so that an individual, translucent silicone index was obtained, which was cut to size and divided horizontally (Figs 4j to 4m). After checking the accuracy of fit on the model, a drainage channel for pressure release was created at the coronal portion of the splint (Fig 4n to 4p).

#### Intraoral fabrication of the composite cantilever restoration with silicone index

Before placing the silicone index, the abutment tooth was cleaned. For reasons of better visibility and control, phosphoric acid was now applied to the tooth surface for 30 s, followed by careful rinsing and drying (Figs 4q and 4r). The basal part of the flexible splint (silicone index) was then placed in the patient’s mouth and checked again for accuracy of fit (Figs 4s and 4t). Since the isolation by means of the silicone index appeared to be sufficient, no further application of rubber-dam was necessary, and the process was continued under relative isolation.

After application of primer and adhesive (Optibond FL, Kerr) with subsequent light curing, flowable composite (Tetric Evo Flow, Ivoclar Vivadent) was filled into the spaces between the splint (silicone index) and the tooth surface and overlaid with a small amount of a conventional packable composite (Tetric Prime, Ivoclar Vivadent; dentin translucency) according to the snowplow technique^7^ without prior light curing (Figs 5a to 5c). Pluggers (Fig 5d) of varied sizes were used to insert the material, as well as a modeling instrument (Optrasculpt). Since the splint was transparent, the placing process could also be seen from the side. Care was taken not to reach the upper margin to ensure the correct fit of the upper part of the splint (Fig 5e and f). This was followed by successive filling of the basal portion of the splint with composite in dentin translucency, with the exception of the buccal surface, which was filled with composite in enamel translucency (Fig 5 g). Light curing was initially performed for 20 s per layer or longer (depending on the layer thickness, see also Table 2) from all sides with the upper part of the splint in place (Fig 5h). In the next step, the coronal part of the splint was filled with enamel-transparent composite (Fig 5i) and, after applying a small, non-light-cured flowable composite layer (Fig 5j), it was placed and pressed down (Fig 5k and l). After light curing (again from all sides for at least 20 s each), the splint was removed. The drainage channel was filled with composite (Fig 5m). Subsequently, finishing and polishing was carried out (Fig 5n to 5p). Oscillating diamonds (Sonic Set shape and Endo-Sonic-Access, Komet, Gebr. Brassler; Lemgo, Germany) (Table 2) were used at the transition from the pontic to the abutment tooth. The pontic created a passage for interdental brushes. Suitable sizes were selected (Fig 5q). The materials used are listed in Table 2. Figures 5r and 5s show the clinical and radiographic control with the pontic in-situ. The patient was able to clean the restoration well and was satisfied with the treatment outcome. Figures 5t to 5w show the condition at the 6-month check-up. The findings (including periodontal parameters) were unremarkable.

### Method 3: Indirectly Fabricated Restorations (Fabricated on a Plaster Model)

**Fig 6:**
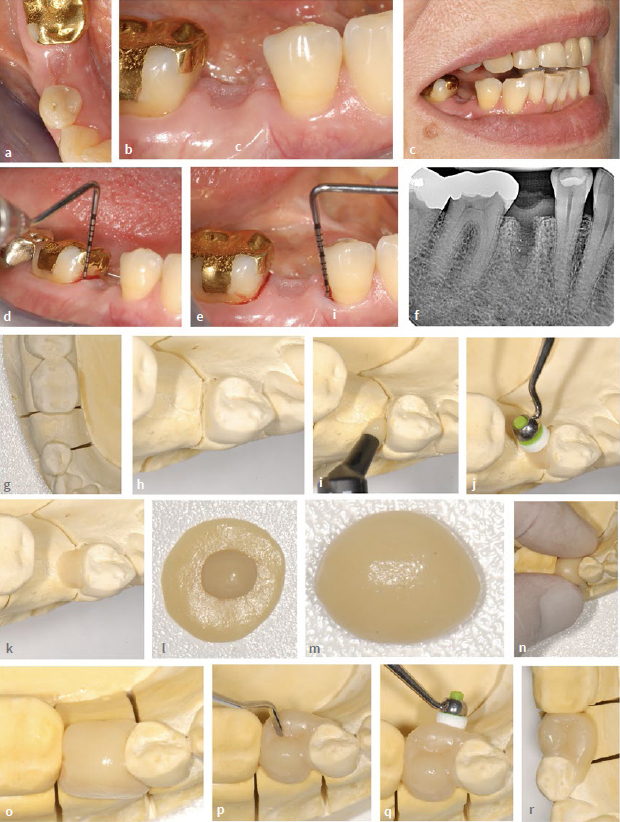
Indirectly (extraorally) fabricated workpiece (modeled on the plaster model) using the example of a category II NPB in a 50-year-old female patient. (a) to (c) Initial situation. (d) and (e) Bleeding on probing. (f) X-ray with the alveolus still visible after recent tooth extraction. (g) Initial situation showing the gap in the region of the lower right second premolar. (h) to (k) Initial application and distribution of composite on the distal surface of tooth 44 (no light curing for the time being!). (l) Composite “ball” (dentin translucency) and composite “plate” (enamel translucency). (m) Ovoid composite structure with “dentin core” and “enamel shell”. (n) and (o) Insertion of the soft composite material into the gap. (p) to (r) during and after modeling. Subsequent light curing (before and after removal from the model).

**Fig 7:**
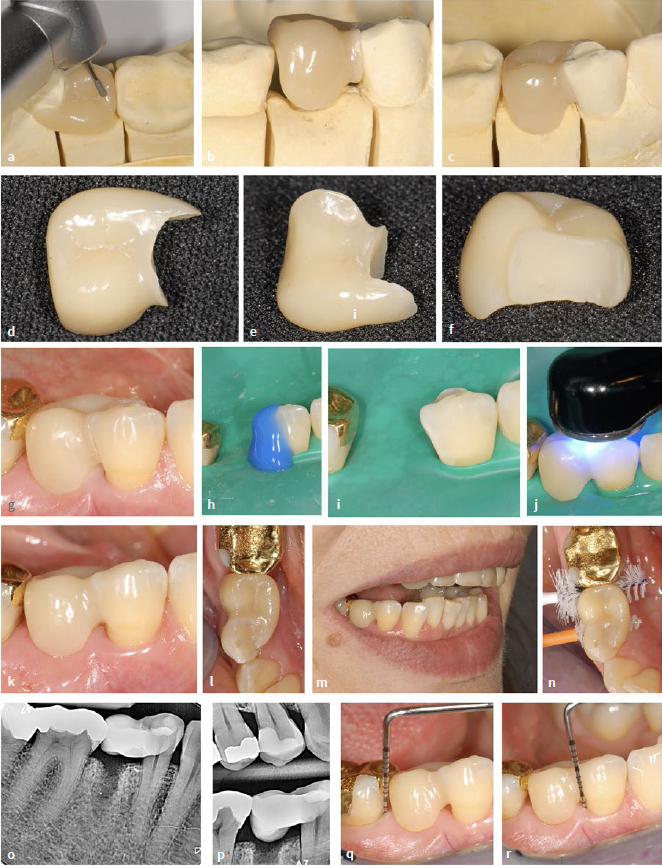
Same patient as in Fig 6. (a) Finishing and polishing on the model. (b) and (c) The distal surface of the pontic is shaped so that the workpiece can be “snapped” into place with the clearest possible position. (d) and (e) Finished and polished workpiece. (f) After blasting the adhesive surface with aluminum oxide powder. (g) Try-in on the patient. If positioning is not clear, an individual fixing splint can be used (see Fig 9o). (h) to (j) Etching, rinsing and drying of the abutment tooth under rubber-dam and adhesive placement with subsequent light curing. (k) After adhesive placement. (l) Note the selective oversizing in the area of the oral surface of tooth 44. (m) Overview. (n) Fitting of suitable interdental brushes. (o) and (p) Radiographic control. (q) and (r) Control after 4 months, no pathological findings, disappearance of bleeding on probing.

In the case described here (Figs 6 and 7), a category II composite cantilever restoration (Fig 1) was used to close the single-tooth gap. For this purpose, the composite pontic was prefabricated by a dental technician on the plaster model of the intraoral situation. The patient was a 50-year-old female who was dissatisfied with her appearance due to a gap in the region of the mandibular right second premolar (Figs 6a to 6c). The general medical history was unremarkable, as were the clinical findings with regard to dental hard tissues, existing restorations, endodontic, periodontal and functional parameters. The periodontal examination did not reveal any increased probing depths, but there was bleeding on probing adjacent to the single-tooth gap (Figs 6d and 6e). The radiograph showed the alveolus of the tooth recently extracted due to a root fracture (Fig 6f).

#### Contouring of the restoration on the model

Alginate impressions were used to fabricate plaster models of the maxilla and mandible (Fig 6 g). Restorative composite was applied to the distal surface of the mandibular right first premolar after isolation of the model. For isolation, vaseline was applied in a very thin layer, then carefully removed to avoid polymerisation inhibition of the composite (Figs 6h to 6k). Light curing of the composite material was not performed. The pontic was formed from a small sphere of resin composite in dentin translucency that was coated with resin composite in enamel translucency (Tetric Prime, Ivoclar Vivadent) (Figs 6l and 6m). The uncured composite was carefully introduced into the gap (Figs 6n and 6o). This was followed by shaping with Heidemann spatulas and a special modeling instrument (Optrasculpt) (Figs 6p to 6r). The connector area was slightly oversized vestibularly and greatly oversized lingually. Now, initial light curing was performed on the model. After removing the pontic from the model, light curing was performed from all sides. This was followed by finishing and polishing (including the basal surface of the pontic) (Fig 7a). During these steps, the distal surface of the pontic was formed in such a way that it could be inserted with a slight “snapping” effect (Figs 7b and 7c). The workpiece showed secure and clear positioning. Figures 7d and 7e show the pontic after completion of the polishing procedure.

#### Preparatory measures and bonding of indirect restoration

**Fig 8:**
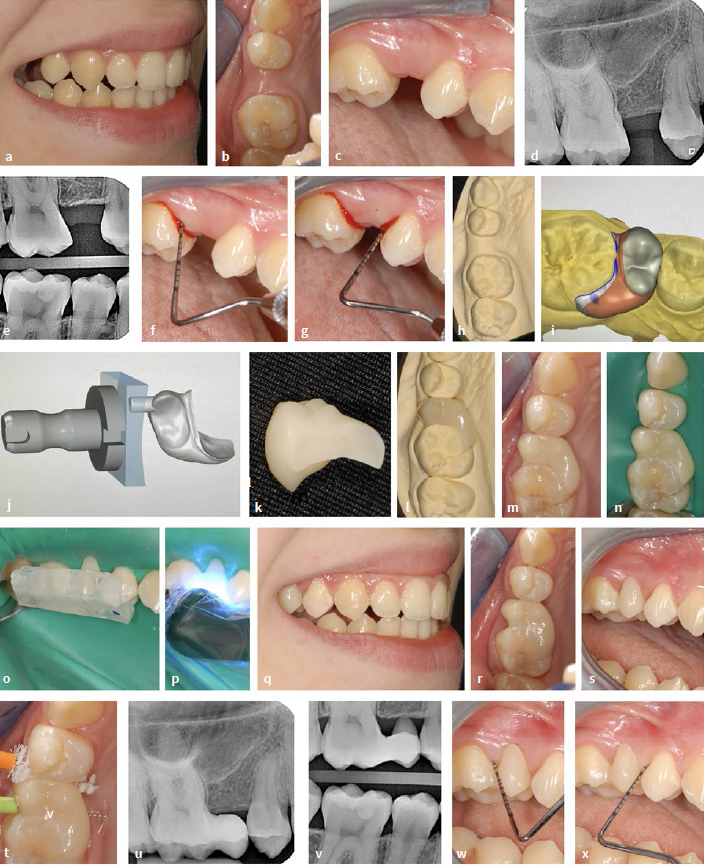
Indirectly (extraorally) fabricated workpiece (digitally fabricated) using the example of a category II NPB in a 21-year-old female patient. (a) to (c) Initial clinical situation with gap (upper right second premolar) of premolar width. (d) and (e) Initial radiographic situation. (f) and (g) bleeding on probing on the tooth surfaces facing the gap. (h) Plaster model. (i) to (k) Digital design of the pontic. (l) Try-in on the model. (m) Try-in in the mouth. (n) Try-in under rubber-dam. (o) Adhesive insertion with individual fixation key (placing guide). (p) Light curing. (q) to (s) Clinical situation after gap closure. (t) Try-in of matching interdental brushes. (u) and (v) Radiographic situation after gap closure. (w) and (x) Check after 2 months, no pathological findings, no bleeding on probing.

Figure 7f shows the adhesive surface after air abrasion with alumina powder (grain size 50 µm). Figure 7g demonstrates the intraoral try-in on the patient and the good fitting accuracy. For this reason, a fixation aid (cf. Fig 8o) was not required in this case. Adhesive insertion was carried out under rubber-dam isolation after etching the tooth surface (mandibular left first premolar) with phosphoric acid (Fig 7h and i). The required primers (Panavia V5, Kuraray Noritake; Tokyo, Japan) were applied to the bonding surfaces of the abutment tooth and the workpiece according to the manufacturer’s instructions. Subsequently, a dual-curing composite cement (Panavia V5, Kuraray Noritake) was applied. The light-curing process is shown in Fig 7j. The materials used can be found in the material list (Table 2). Oscillating diamonds (Sonic Set shape and Endo-Sonic-Access, Komet; Table 2) were used at the transition from the pontic to the abutment tooth. Figures 7k to 7m show the situation after adhesive placement. Figure 7n demonstrates the selection of suitable interdental brushes. Figures 7o and 7p show the radiographic control.

A check-up after 4 months revealed no pathological findings, and the initial bleeding had disappeared after this brief period of time (Figs 7q and 7r). The patient was very satisfied with the situation.

### Method 4: Indirectly Fabricated Workpieces (CAD/CAM)

**Fig 9:**
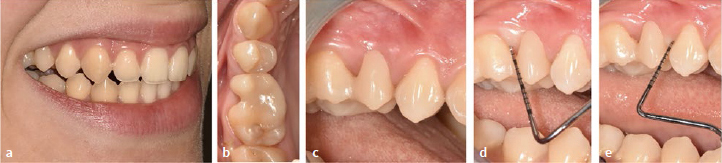
Same patient as in Fig 8. (a) to (e) At the 6-month check-up. Inconspicuous clinical situation including periodontal conditions, disappearance of bleeding on probing.

In the case described here (Figs 8 and 9), a category II composite cantilever restoration (Fig 1) was also used to close a gap of premolar width. For this purpose, a workpiece was digitally designed and manufactured from a prepolymerized block (Tetric CAD; for further product selection, see material list and discussion) using CAD/CAM technology. The patient was a 21-year-old female who was dissatisfied with her appearance because of the gap in the region of the maxillary right second premolar (Figs 8a to 8c). The general medical history did not reveal any special features. The clinical findings regarding dental hard tissues, existing restorations, endodontic, periodontal and functional parameters were largely without pathology. Radiographically, the situation was unremarkable (Figs 8d and 8e). Periodontal examination of the abutment teeth revealed slightly increased probing depths and bleeding after probing as a sign that the patient was only partially able to control plaque on the tooth surfaces adjacent to the gap (Figs 8f and 8 g).

#### Digital scan, design and manufacturing

Alginate impressions were used to produce plaster models of the maxilla and mandible (Fig 6h), which served as the basis for the digital design. The model of the maxilla was also used to custom-fabricate a placing guide (see below). A digital intraoral impression would have been possible, but was not taken in this case. The fabrication can be divided into five design phases. After the restoration type was determined (1), the digital scan was performed with the Prime Scan AC (2). In the subsequent modeling phase (3), design limits were calculated, including the definition of margins and axes. This was followed by the design phase (4) and finally the fabrication phase (5). The software used was Cerec SW 524 and the milling unit Cerec Primemill (both Dentsply Sirona; Konstanz, Germany). Figures 8i to 8k show the digital design and the finished restoration.

#### Preparatory measures and bonding of indirect restoration

In order to ensure proper placement, a placement guide was created. The restoration was positioned optimally on the model and surrounded with a silicone compound. Once it solidified, it was carefully trimmed to ensure proper alignment with the occlusal surfaces of the neighboring teeth and the NPB. It is important to note that this placement guide served a different purpose than the silicone index mentioned in method 2. Figure 8l visualizes the try-in on the model, Fig 8m in the patient’s mouth before rubber-dam was applied, and Fig 8n after rubber-dam was applied. Air abrasion was performed on the bonding surface of the restoration with alumina (50 µm). The surface was then cleaned and moistened with the designated primer (Panavia V5, Kuraray Noritake; without light curing) according to the manufacturer’s instructions. After cleaning, the mesial surface of maxillary right first molar was conditioned with phosphoric acid for 30 s, rinsed, dried, and touched with a primer. A dual-curing adhesive and composite cement (Panavia V5, Kuraray) was used. Once the restoration was inserted, the placement guide was positioned and pressed firmly against the abutment tooth using a Heidemann spatula, providing clear and precise alignment (Fig 8o). This was followed by light curing from all sides and finishing (Fig 8p). The materials used can be found in the materials list (Table 2). For finishing, oscillating diamonds (Sonic Set shape and Endo-Sonic-Access, Komet) (Table 2) were used at the transition from the pontic to the abutment tooth. Figures 8q to 8s demonstrate the clinical situation after gap closure. Figure 8t shows the try-in of interdental brushes. The radiographic controls (Figs 8u and 8v) indicate a continuous transition of the composite cement from the restoration to the tooth. A first check-up after 2 months revealed no pathological findings, and the initial exploratory bleeding had already disappeared after this brief period of time (Figs 8w and 8x). The patient coped very well with the restoration. Figures 9a to 9e (same patient as in Fig 8) show the condition at the 6-month check-up. The findings (including periodontal parameters) are without pathology.

## Discussion

The basis for deciding if and how a gap should be closed is variable and sometimes not clearly justified.^6^ Currently, implants and conventional fixed partial dentures are often considered the first choice.

Promising innovations regarding adhesive fixed partial dentures have been presented recently.^5,25^ Yazigi and Kern^25^ were able to show that cantilever fixed partial dentures made of all-ceramic materials are a durable treatment option in the posterior region. However, in comparison to the methods presented in this case series, they still require certain preparations (including the occlusal surface area), although to a reduced extent. However, the designs described by Yazigi and Kern with delicate occlusal, vestibular and oral ceramic extensions^25^ are not feasible with composite materials, as the latter have a lower fracture resistance than ceramics. For this reason, the use of composite materials relies on larger dimensions, which requires selective oversizing according to the design described in 2019.^12^

Non-invasive procedures for single-tooth replacement in the posterior area were introduced starting in the 1990s.^9^ Various clinical and experimental studies followed, with indications regarding the practical procedure.^12-15^ This work also shows the historical development of NPBs with their various categories and their technical refinements.

In 2015, success rates of non-invasive composite restorations for posterior single tooth gaps were reported for the first time in a clinical study in the international literature.^10^ This study was still exclusively concerned with category I composite buildups (Fig 1). In 2021, a more comprehensive clinical study was published^19^ that included the categories I to V (up to molar width, Fig 1). The observation period ranged from one to 21.5 years, with longer observation periods for category I restorations (Fig 1) than for the more recently presented categories II to V (Fig 1). Three non-invasive restorations failed (cohesive fracture and/or adhesive failure), 50 of total of 53 non-invasive restorations were intact, and the periodontal situation also remained largely inconspicuous.^19^ In order to avoid periodontal problems despite the oversized restorations, regular instructions (usually once a year) on the correct selection and handling of interdental brushes took place.^8,16^

The observations show that fractures (cohesive fracture and/or adhesive failure) are a weak point. Potential fracture risks and general measures of prevention of fractures are summarized in Table 3. In this context, special attention should be paid to functional aspects, taking into account the occlusion and articulation of the teeth. Missing or unsupported teeth in the posterior region, poor mastication and bruxism can be contraindications. In principle, the three new methods (2 to 4) described in this paper correspond to the restorations previously investigated with method 1 (non-invasive, cantilever design, selective oversizing in the palatal/lingual connector area, usually consisting of resin composite only). The differences lie only in technical measures to simplify the procedure (direct intraoral insertion with individual “shaping aids” according to method 2, insertion of indirectly fabricated composite restorations according to methods 3 and 4).

**Table 3:** Potential fracture risks and general fracture prophylaxis measures

Risks of potential fractures	Prevention of fractures
– Low physiological mobility of the abutment tooth – High masticatory forces in the posterior region (incl. bruxism) – Sharp antagonist cusps – Limited adhesion surfaces (e.g., short crowns) – Rigid blocking on both sides – Processing errors during adhesive placement	– Use of maximum bonding surface (incl. vertical dimensions) of the abutment tooth – Use of selective oversizing (especially palatally/lingually) – Rounding of antagonist cusps if necessary – Avoiding eccentric contacts – Preferably cantilever design – Correct adhesive placement under tension-free rubber-dam


In method 2, newly developed “shaping aids” (silicon indices) are used. After insertion of the basal “splint” part, and if necessary, further isolation measures, the composite is inserted in several steps, finishing with the coronal part of the “splint”. The procedure differs from similar applications described for other purposes, such as the injection of flowable composites for restorative purposes^1,2,21^ or for acrylic splints.^20^

Methods 3 and 4 use indirectly fabricated restorations. Preparation of the teeth with special grooves and recesses as a prerequisite for the exact placement of the restorations is not necessary, as this is also possible by other means (e.g., with placing guides) (cf. Fig 8o).

In method 3, the restorations are made of light-cured composites, which are commonly used in restorative therapy. Their bonding surfaces can be roughened very well by alumina air abrasion, among other things, so that the risk of debonding between the composite cement and the restoration is reduced – similar to repaired restorations.^22^ The situation is different for method 4. According to the literature, debonding rates were so high in some industrially cured composite blocks that some products had to be withdrawn from the market.^3^ The literature does not yet contain precise information on which adhesive bonds are required for non-prep bridges. It would be desirable to have a product range of CAD/CAM composite blocks that, after air abrasion, show the same surface roughness as conventional light-cured composite. An alternative is the use of composite-ceramic combinations (hybrid-type, polymer-infiltrated ceramics), whose bonding surfaces can be conditioned by hydrofluoric acid etching followed by silanization.^4^ In-vitro studies have shown that this increases the bond strengths to composite.^3^

To date, the above methods have been used to close single-tooth gaps up to one molar width, with a pontic not exceeding premolar width. As far as abutment selection is concerned, various components (anatomy and surface morphology of the abutment teeth, periodontal condition, axial position or tilting, shape of the alveolar ridge, esthetic requirements, etc) must be considered. However, there are no studies so far that allow clear statements which of the procedures mentioned can be expected to have the highest success rate (see below for further explanations).

All four methods presented here have advantages and disadvantages, which are listed in Table 4. The following can be stated regarding method selection (see also Table 5):

**Table 4:** Advantages and disadvantages of different methods for the fabrication of non-prep bridges

	1. Direct intra-oral composite insertion without individual shaping aids (“splints”)	2. Direct intra-oral composite insertion with individual shaping aids (“splints”)	3. Indirectly (extra-orally) fabricated restorations (modeled from composite)	4. Indirectly (extra-orally) fabricated restorations (created digitally)
Degree of difficulty for dental work	High	Easier than method 1	Easier than methods 1 and 2	Easier than methods 1 and 2
Time required by patient	High	Less than method 1	Less than method 1 and 2	Less than method 1 and 2
Dental technician	Not required	Required (model preparation, splints)	Required (model preparation, direct modeling and fabrication of the restoration on the model, fabrication of a place guide if necessary)	Required (conventional and/or digital model preparation, digital design and fabrication of the restoration, fabrication of a place guide if necessary)
Interface (tooth surface/composite)	Direct application of composite to the natural or restored surface of the abutment tooth, only one interface is created (tooth surface/composite)	Only one interface is created (between natural or restored tooth surface and composite), the fit of the silicone index (“splint”) has no influence on the interface	A double interface is created (tooth surface/composite cement/composite workpiece), which can be disadvantageous if the restoration does not fit exactly; debonding risk	A double interface (tooth surface/composite cement/composite workpiece) is created, which can be disadvantageous if the restoration does not fit exactly; debonding risk
Finishing, polishing	Requires easy access	Requires easy access	Usually uncritical	Usually uncritical
Base of pontic	Not polishable	Smooth surface achievable with silicone index, but not polishable	Polishable	Polishable


**Table 5:** Method selection taking into account individual patient requirements

Patient-related factors	1. Direct intra-oral composite insertion without individual shaping aids (“splints”)	2. Direct intra-oral composite insertion with individual shaping aids (“splints”)	3. Indirectly (extra-orally) fabricated restorations (modelled from composite on the model)	4. Indirectly (extra-orally) fabricated restorations (on a digital basis)
Uncomplicated anatomical conditions (e.g., straight tooth axes, regular macrostructure of abutment teeth)	+	+	+	+
Complex anatomical conditions (e.g., tilted tooth axes, irregular macrostructure of abutment teeth)	+	(+)	-	-
Simple shaping possibilities (e.g., good adaptability to the alveolar ridge, easy adaptation to existing occlusion and the dental arch)	+	+	+	+
Complex shaping requirements Complex shaping requirements (e.g., unfavorable alveolar ridge shape, difficult adaptation to existing occlusion and the dental arch)	-	(+)	(+)	(+)
Favorable adhesion surfaces (e.g., large bonding surfaces in vertical and horizontal dimension, teeth without restorations)	+	+	+	+
Limited adhesion surfaces (e.g., small bonding surfaces in vertical and horizontal dimensions, teeth with existing restorations of variable size and composition)	(+)	(+)	(+)	-

+ = feasibility usually given; (+) = feasibility with restrictions; - = feasibility critical.

- Direct intraoral composite insertion without individual shaping aids (method 1) can be considered for both inconspicuous and difficult anatomical conditions (e.g., tilted axes, irregular tooth surfaces). However, there should be a manageable access to the area in question. In addition, patients must be fit for longer treatment sessions.
- Direct intraoral composite insertion with individual shaping aids (method 2) is also possible for most anatomical conditions. However, the situation can be visualized beforehand on the plaster model and the treatment session can be kept shorter than with method 1.
- Indirectly (extraorally) fabricated restorations which are shaped manually (method 3) allow maximum utilization of the bonding surface due to better visualization, which is particularly important with limited bonding surfaces (e.g., short crowns). However, they require uncomplicated anatomical conditions (e.g., straight tooth axes, regular tooth surfaces) for an easy insertion.
- Digitally fabricated restorations require the largest possible bonding surfaces (e.g., rather long dental crowns), since the full utilization of the bonding surface in the vertical dimension is not always possible due to CAD/CAM limitations. They also require uncomplicated anatomical conditions.

In summary, the four methods can be used to cover numerous clinical situations. Further experimental and clinical studies are underway to provide more scientifically validated recommendations for case selection. New interdisciplinary perspectives with additional strategies and options for non-invasive replacement of single missing teeth in the sense of “Frugal Dentistry” are emerging for the future.

### Frugal Dentistry

The current state of knowledge on Frugal Dentistry was published in 2017 and 2021.^11,17^ Frugal interventions are defined by the following three main criteria:

- Focus on core functionalities, taking into account dental standards.
- Optimized level of service, taking into account the expectations and needs of the people to be addressed (demand).
- Substantial cost reduction (modified after Weyrauch).^23^

The eventful history of dental acrylics and adhesives can be used as an example to show that this group of materials, which was introduced into prosthetic dentistry as early as 1930, is particularly relevant for frugal innovations.^18^ The composite resins introduced later have also repeatedly led to “frugal” surprises in restorative dentistry.

The strengths of the methods (non-invasiveness, cost-effectiveness, etc) are currently offset by weaknesses (limited practical experience, few scientific studies, etc).

At this stage, NPBs are still partly experimental. They can be successfully performed primarily by dentists who already have extensive experience with complex direct restorations. Several prerequisites, such as absence of bruxism and good oral hygiene, must also be considered when selecting patients. The ability to perform dental hygiene at the abutment tooth is a crucial factor, which applies not only to indirect restorations but also when removing hard-to-reach excess luting composite. Improper execution of this process can potentially lead to the development of caries or periodontal pockets in otherwise healthy abutment teeth. Patients need to be informed about these risks and the importance of regular check-ups to uphold the principles of preventive and non-invasive dentistry.

When evaluating the fit of indirect restorations on non-prepared abutment teeth (methods 3 and 4), proper positioning becomes crucial along with the assessment of gaps between the tooth and the restoration. Although gaps are typically inconspicuous with both methods, achieving precise positioning appears to be easier with method 3 than method 4. This sometimes necessitates the use of placement guides, as described in the Results section.

The time required for the procedures varies depending on the initial conditions and the dentist’s level of experience. Method 1 generally takes around two hours. Method 2 tends to be slightly shorter due to the assistance of a silicone index in shaping the restoration. With methods 3 and 4, the dentist does not need to fabricate the restorations. However, the working steps involved are demanding and complex, including preparation of the working area, accuracy checks for fit and positioning, adhesive insertion, finishing, occlusion and articulation checks, and hygiene assessment. Therefore, a significant amount of time is also required for these methods. Considerable efforts are still needed to promote the further development of non-prep bridges (NPBs). In addition to a desirable improvement of the material properties of the restorative materials used, this work focusses on improved fabrication, insertion and finishing techniques. In the future, it will be crucial to generate more in-vitro and in-vivo studies on these new restoration types.

## Conclusions

The present case series shows that technical improvements for the closure of single tooth gaps are possible through various innovations. On the one hand, there are new options through splint-assisted direct insertion techniques. On the other hand, conventionally or digitally manufactured restorations with special designs provide a promising contribution to the simplification of the working steps. Stable and hygienic restorations with composite without tooth preparation (non-prep bridge, NPB) are now feasible, but require comprehensive expertise in the field of conservative-restorative dentistry. The (few) clinical retrospective evaluations available to date have demonstrated good survival rates over extended periods of time. The establishment of a non-invasive, esthetically pleasing, stable and affordable restoration to treat single-tooth gaps as a supplement or alternative to previous interventions can also serve as an important component of prevention-oriented dentistry.

## Clinical relevance:

The non-invasive and cost-effective closing of single-tooth gaps contributes to the preservation of oral structures and opens up new perspectives for Frugal Dentistry.
